# Apical Closure of Immature Molar Roots: A Rare Case Report

**DOI:** 10.5005/jp-journals-10005-1010

**Published:** 2008-12-26

**Authors:** Deepti A, Shifa S, Muthu MS, Rathna Prabhu V

**Affiliations:** 1 Senior Lecturer, Department of Pediatric Dentistry, Meenakshi Ammal Dental College, Alapakkam main road Maduravoyal, Chennai, India; 2 Postgraduate Student, Department of Pediatric Dentistry, Meenakshi Ammal Dental college, Alapakkam main road, Maduravoyal, Chennai, India; 3 Professor, Department of Pediatric Dentistry, Meenakshi Ammal Dental College, Alapakkam main road, Maduravoyal, Chennai, India; 4 Professor and Head, Department of Pediatric Dentistry Meenakshi Ammal Dental College, Alapakkam main road, Maduravoyal, Chennai, India

**Keywords:** Molar apexification, Calcium hydroxide, Immature permanent molar roots, Pulpdent

## Abstract

This is a rare case report of apexification in an immature permanent mandibular first molar. Calcium hydroxide was used for apical root closure of both the mesial and distal canals. Root closure occurred after 13 months following which obturation of the tooth was completed.

## APICAL CLOSURE OF IMMATURE MOLAR ROOTS—A RARE CASE REPORT

The treatment of incompletely formed pulpless teeth has presented considerable problems. These teeth have wide open apexes and the walls of the root canal diverge toward the apical tissues. Mechanical preparation cannot be done in the normal manner because of the large initial size and the flare of the apical part of the canal. A mechanical stop cannot be produced at the apex of the canal and, therefore, control of root filling materials is difficult. Pulpless teeth with open apexes sometimes have been treated by cleaning the canal, overfilling with a standard root filling material, and then performing a modified root resection. This stops root formation, and the result is a short, weak tooth with a doubtful prognosis.^1^

Apical root closure may result from apexification or bridge formation and is indicated when the pulps of young permanent teeth become necrotic. Various techniques were used to induce the apexification process. Cooke and Rowbotham^2^ reported a ten-year follow up of incompletely formed pulpless teeth that they had treated by dressing the canals with tricresol and formalin. The canals were subsequently filled with a paste of zinc oxide and eugenol containing iodoform and thymol. They were able to show radiographically that further root formation had occurred after the root treatment.

Kaiser^3^ obtained apexification with calcium hydroxide paste. Frank^4^ and Heithersay^5^ obtained successful results when calcium hydroxide in various forms was introduced in canals of immature teeth. Cvek^6^ examined the results of treating 55 maxillary incisors with immature roots, complete pulpal necrosis, and radiographically demonstrable periapical tissue changes. After antibiotic treatment the canals were filled temporarily with calcium hydroxide. Radiographic bone healing and apical closure were noted in 50 teeth. Steiner and Van Hassel^7^ did histological studies and reported the formation of cementum like hard tissue after treatment with calcium hydroxide combined with camphorated monochlorophenol. Dylewski^8^ stated that the calcified material that forms at the apex resembles osteodentin. Mineral trioxide aggregate, a newer material is a suitable replacement for calcium hydroxide for the apexification in immature roots.^9^ Recently a new technique was introduced wherein revascularization of the necrotic infected pulp space of an immature permanent maxillary central incisor was induced in-vivo by stimulation of blood clot from the periapical tissues into the canal space.^10^

Pulpdent root canal sealer is an ADA accepted permanent root canal filling material. It can be used in both primary and permanent teeth. It is tissue compatible, bacteriostatic, radiopaque and insoluble in the root canal. It sets in two to three hours and it does not shrink upon setting ensuring a positive seal. In the early 1960s, Greenberg and Katz^11^designed the endodontic pressure syringe, which was manufactured by the Pulpdent Corporation. It is a simple and accurate method of obturating the root canal space. It eliminates voids and also the problems associated with inaccessibility of some posterior teeth having narrow or tortuous canals.

Apexification procedures are most often undertaken in immature permanent anterior teeth where as few case reports in premolars1^2^ and primary anterior teeth1^3^ are also present. A PUBMED and a MEDLINE search did not reveal any case of apexification of immature permanent molar. Rotstein, Friedman and Katz1^4^ reported 2cases in which calcium hydroxide induced apical root closure in posterior mature teeth where the apical constriction was lost because of chronic inflammatory process. Weine1^5^ reported apexification in the palatal root of a maxillary molar where previous caries followed by failed vital pulp therapy left the palatal root undeveloped. Hence this is a rare case report of an apexification in immature mandibular permanent molar.

## Case Report

**Fig. 1:**
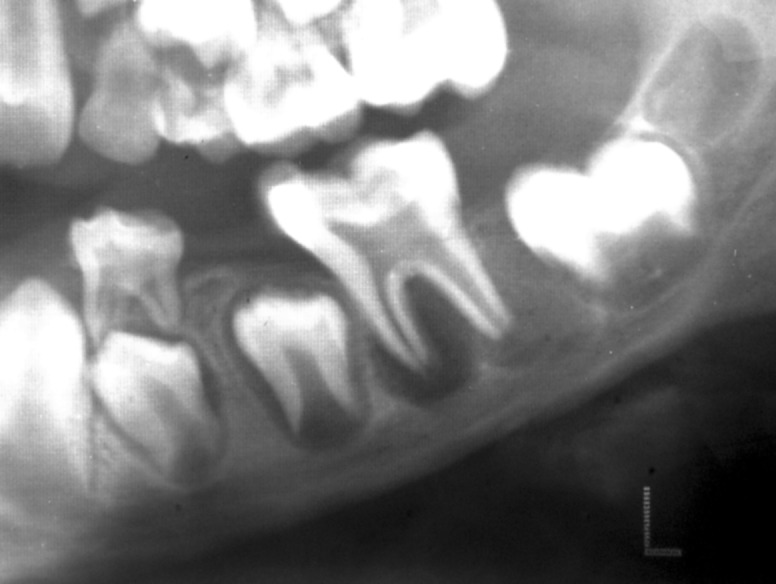
Preoperative radiograph revealing periapical radiolucency in relation to incompletely formed mesial and distal roots in tooth #19

**Fig. 2:**
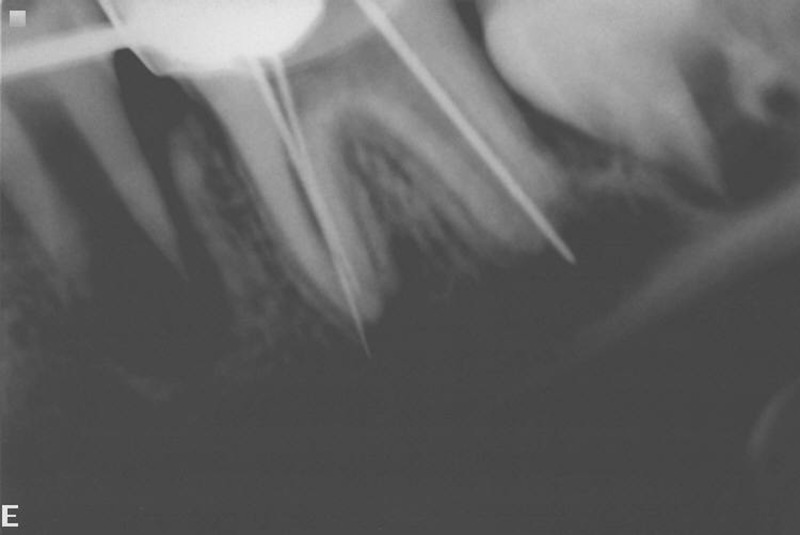
Radiograph showing apex not closed in one of the mesial and distal canals

**Fig. 3:**
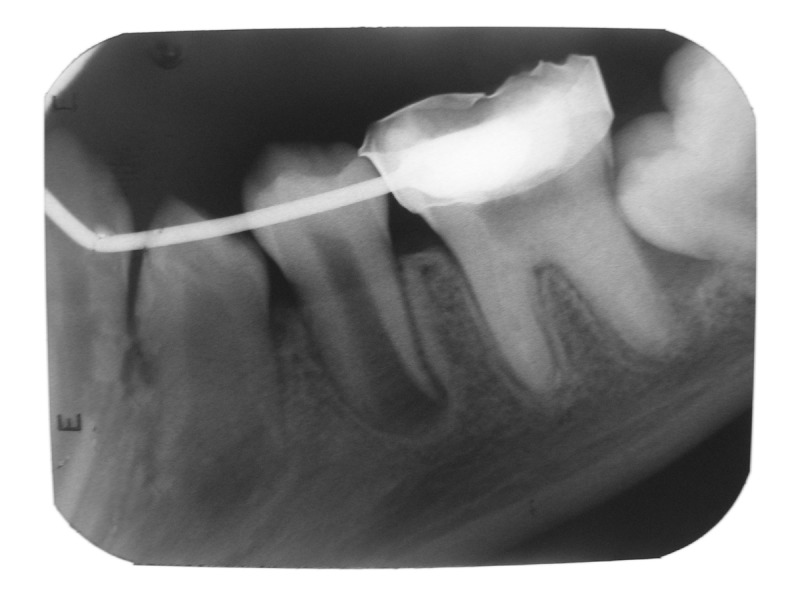
Apexification with calcium hydroxide done in mesial and distal roots with resolution of the periapical radiolucency

**Fig. 4:**
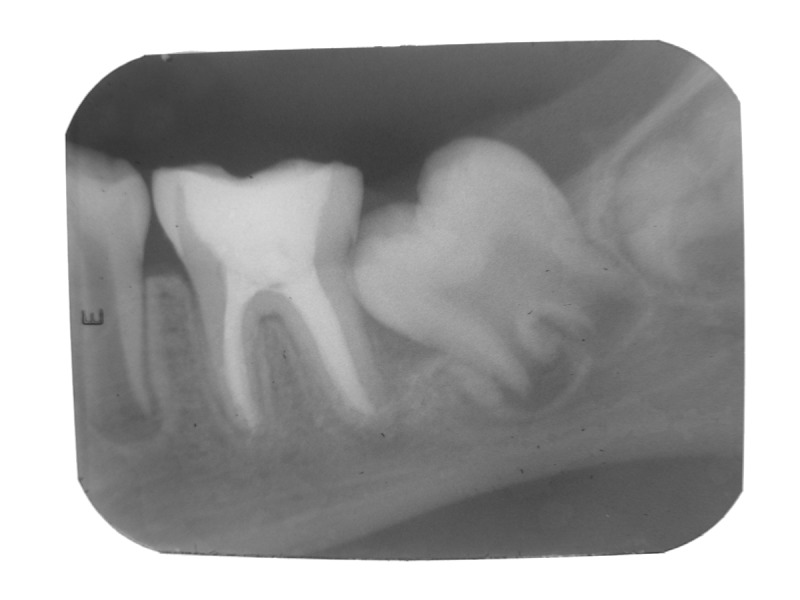
Obturation done with pulpdent root canal sealer

A 10-year-old boy reported to the department with the chief complaint of intermittent pain in the lower left posterior tooth region which was of one month duration. Past medical and past dental history was non-contributory. Clinical examination revealed dental caries in left mandibular first permanent molar (tooth #19). The tooth was found to be tender on percussion. Radiographic examination revealed periapical radiolucency in relation to incompletely formed mesial and distal root apices in tooth #19 (Fig. 1). After isolation, access opening was done and the necrotic pulp tissue was removed with a large barbed broach. Working length was determined slightly short of the radiographic apex. Instrumentation was performed with a gentle circumferential filling motion, aided by copious irrigation with saline. Large sterile paper points were used to dry the canals. The canals were filled with calcium hydroxide paste in a premixed syringe (Calcicur–Voco-Cuxhaven-Germany). The tooth was then temporarily restored with glass ionomer cement. Immediate post operative radiograph was taken which confirmed the presence of calcium hydroxide paste in the canals. Patient was asked to report after three months for review but patient returned only after ten months. Radiographic examination revealed resolved periapical radiolucency and satisfactory apical closure in one of the mesial canal. The other mesial canal and the distal canal did not have satisfactory apical closure (Fig. 2). Hence apexification procedure was repeated by placing calcium hydroxide paste as previously mentioned (Fig 3). A hard apical barrier was detected radiographically with tactile sensation using a 15 size K file after 3 months. Working length was determined and cleaning and shaping of the canals were done upto 40 size using K files. Obturation was accomplished with pulpdent root canal sealer using endodontic pressure syringe. Access cavity was restored with glass ionomer cement (Fig. 4). Post endodontic restoration was done.

## Discussion

The most important factors for apexification are canal cleaning, in other words removal of all necrotic tissue, and the temporary hermetic sealing of the tooth to avoid bacterial infiltration. Diverse materials have been proposed to induce the apexification of nonvital permanent teeth such as zinc oxide- iodoform,^2^ polyantibiotic paste,^16^ Walkhoff’s antiseptic paste,^17^ calcium hydroxide based materials^3^-^8^ resorbable tricalcium phosphate,^18^ Vitapex,^19^or without any inducement material at all^20^. The most commonly advocated medicament is calcium hydroxide, although recently considerable interest has been expressed in the use of MTA.^9^

The biological and bacteriological actions of calcium hydroxide, which was introduced in dentistry by Hermann in 1930^21^, confirm its preference for intracanal use. Kaiser^3^ and Frank^4^ were the first to use it for reliable closure of immature roots. Since then, root closure of pulpless permanent teeth with calcium hydroxide has become an accepted procedure and is well documented in literature. Morse et al^22^ studied 5 treatment methods for teeth with incomplete root formation and pulpal necrosis and concluded that the success of therapy with apical tissue repair is due to the antibacterial action and the calcification-inducing action of calcium hydroxide. Selden^23^ in permanent mandibular cuspid. Following apexification, the root end morphologically closely resembled normal root end formation despite the evidence of total pulal necrosis and infection. According to Sheehy and Roberts 24 the use of calcium hydroxide for apical barrier formation is successful in 74-100% of cases irrespective of the proprietary brand used. Hence in our case we decided to use calcium hydroxide paste for apexification.

Apical hard tissue barrier induced by either apexification or apexogenesis is usually irregular and porous and may take the form of a cap or a bridge. Histologically, its characteristics may be of dentin, cementum or osteodentin.^7^,^8^ Its formation may take 3 to 24 months.^25^ Gupta, Sharma and Dang^26^ conducted a single visit apexification in a non-vital and immature mandibular premolar and concluded that frequent changing of the calcium hydroxide dressing is not always required to induce apical closure. In our case a barrier was found to be formed at 13 months following apexification.

## References

[1] Binnie WH, Rowe AHR (1973). A histological study of the periapical
tissues of incompletely formed pulpless teeth filled with calcium
hydroxide. J Dent Res.

[2] Cooke C, Rowbotham TC (1960). Root canal therapy in non vital teeth with open apices. Br Dent J.

[3] Kaiser JH (1964). Presentation to the American Association of
Endodontists..

[4] Frank AL (1966). Therapy for divergent pulpless tooth by continued
apical formation.. J Am Dent Assoc.

[5] Heithersay GS (1970). Stimulation of root formation in incompletely
developed pulpless teeth. Oral Surg Oral Med Oral Pathol.

[6] Cvek M (1972). Treatment of non-vital permanent incisors with calcium hydroxide. I Follow-up of periapical repair and apical closure of immature roots. Odontol Revy.

[7] Steiner JC (1971). Van Hassel HJ. Experimental root apexification in
primates.. Oral Surg Oral Med Oral Pathol.

[8] Dylewski JJ (1971). Apical closures of non-vital teeth.. Oral Surg Oral Med Oral Pathol.

[9] El-Meligy OA, Avery DR (2006). Comparison of apexification with mineral trioxide aggregate and calcium hydroxide. Pediatr Dent.

[10] Thibodeau B, Trope M (2007). Pulp revascularization of necrotic infected immature permanent tooth: case report and review of the literature. Pediatr Dent.

[11] Greenberg M (1963). Filling root canals by an injection technique. Dental
Digest.

[12] Seo R, Maki K, Hidaka A, Higuchi M, Kimura M (2005). Long term radiographic study of bilateral second premolars with immature root treated by apexogenesis and apexification. J Clin Pediatr
Dent.

[13] Almeida ML, Damasceno LM, Primo LG, Portela MB (2002). Apexification of primary teeth: a treatment option. J Clin Pediatr Dent.

[14] Rotstein I, Friedman S, Katz J (1990). Apical closure of mature molar roots with the use of calcium hydroxide. Oral Surg Oral Med
Oral Pathol.

[15] Weine FS (2004). Endodontic Therapy.

[16] Ball JS (1964). Apical root formation in nonvital immature permanent
incisor. Report of a case.. Br Dent J.

[17] Bouchon F (1966). Apex formation following treatment of necrotized
immature permanent incisor.. J Dent Child.

[18] Koenigs JF, Heller AL, Brilliant JD, Melfi RC, Driskell TD (1975). Induced apical closure of permanent teeth in adult primates using a resorbable form of tricalcium phosphate ceramic.. J Endod.

[19] Lu YM, Qin JN (2004). [A comparison of the effect between Vitapex
paste and antibiotic paste in apexification]. Shanghai Kou Qiang
Yi Xue..

[20] Chawla HS, Tewari A, Ramakrishnan E (1980). A study of apexification
without a catalyst paste. ASDC J Dent Child.

[21] Hermann BW (1930). Dentinobliteration der wurzelkanale nach
behandlung mit kalzium.. Zahnarztl Rundschau.

[22] Morse DR, O’Larnic J L, Yesilsoy C (1990). Apexification: review of the
literature. Quintessence Int.

[23] Selden HS (2002). Apexification: An interesting case.. J Endod.

[24] Sheehy EC, Roberts GJ (1997). Use of calcium hydroxide for apical
barrier formation and healing in non-vital immature permanent
teeth: a review.. Br Dent J.

[25] Bradley HL (1977). The management of the nonvital anterior tooth
with an open apex.. J Br Endod Soc.

[26] Gupta S, Sharma A, Dang N (1999). Apical bridging in association with
regular root formation following single-visit apexification: a case
report.. Quintessence Int.

